# Development and validation of nomograms based on pre-/post-operative CEA and CA19-9 for survival predicting in stage I-III colorectal cancer patients after radical resection

**DOI:** 10.3389/fonc.2024.1402847

**Published:** 2024-10-11

**Authors:** Xuan Dai, Yifan Li, Haoran Wang, Zhujiang Dai, Yuanyuan Chen, Yun Liu, Shiyong Huang

**Affiliations:** ^1^ Department of Colorectal and Anal Surgery, Xinhua Hospital, Shanghai Jiao Tong University School of Medicine, Shanghai, China; ^2^ Department of Gastrointestinal Surgery, Huaihe Hospital of Henan University, Kaifeng, Henan, China; ^3^ The First Clinical School, Xinxiang Medical University, Xinxiang, Henan, China

**Keywords:** CEA, CA19-9, nomogram, colorectal cancer, overall survival, disease-free survival

## Abstract

**Background:**

Carcinoembryonic antigen (CEA) and carbohydrate antigen 19-9 (CA19-9) are the predominant serum tumour markers (STMs) for predicting the prognosis of colorectal cancer (CRC). The objective of this research is to develop clinical prediction models based on preoperative and postoperative CEA and CA19-9 levels.

**Methods:**

1,452 consecutive participants with stage I-III colorectal cancer were included. Kaplan-Meier method, log-rank test, and multivariate COX regression were used to evaluate the significance of preoperative and postoperative STMs. Patients were grouped into a discovery cohort (70%) and a validation cohort (30%). Variables for the nomograms were selected according to the Akaike information criterion (AIC). Subsequently, two clinical predictive models were constructed, evaluated, validated, and then compared with the AJCC 8th TNM stage.

**Results:**

The overall survival (OS) rate and disease-free survival(DFS) rate declined progressively as the number of positive tumour markers(NPTMs) before and after surgery increased. For both OS and DFS, age, sex, pN stage, and NPTMs before and after surgery were independent prognostic factors, and then clinical prediction models were developed. The Concordance index (C-index), Receiver operating characteristic (ROC) curve, calibration curve, Decision curve analysis (DCA), and risk score stratification all indicated that the models possessed robust predictive efficacy and clinical applicability. The Net reclassification index (NRI) and Integrated discrimination improvement (IDI) indicated that the performance of models was significantly superior to the TNM stage.

**Conclusion:**

Nomograms based on pre-and postoperative CEA and CA19-9 can accurately predict survival and recurrence for stage I-III CRC patients after radical surgery, and were significantly better than the AJCC 8th TNM stage.

## 1 Introduction

Colorectal cancer (CRC) ranks as the world’s second most deadly malignancy (1). Despite advancements in surgical techniques and integrated therapies, the clinical outcomes for CRC patients remain unsatisfactory. Approximately fifteen percent of stage II patients and thirty percent of stage III patients experience recurrence even after radical resection (2, 3). The high recurrence and mortality rates have increasingly drawn attention to the need for individualized treatment and prognosis of this disease. Clinicians currently rely on the TNM staging system to predict and assess the prognosis of patients with colorectal cancer (4). While the current staging system provided essential insights into tumour behavioral characteristics, it doesn’t fully encompass vital determinants of patient prognosis, such as age, serum tumour markers(STMs) and so on. Consequently, there’s an imperative demand to unearth novel markers for individualized prognostic assessment, empowering clinicians to offer more precise counsel on survival forecasts and therapeutic approaches for CRC patients.

Owning to its simplicity and cost-effectiveness, tumour marker detection is extensively performed in medical institutions. Carcinoembryonic antigen (CEA) and carbohydrate antigen 19-9 (CA19-9) represent the primary STMs for preoperative evaluation and postoperative follow-up examination of CRC patients. CEA is an acidic glycoprotein associated with oncogenic advancement (5). Some clinical guidelines recommend CEA as a prognostic biomarker for CRC and endorse its routine measurement after radical resection in CRC patients (6, 7). CA19-9 is closely linked to recurrence and survival in colorectal cancer (8). Significantly, combined tumour marker testing has significantly improved predictive accuracy compared with single marker testing (9). Concurrently, the number of positive tumour markers(NPTMs) is gaining attention (10, 11). Previous research has demonstrated its feasibility as a prognostic factor for stage II-III CRC (12). However, while this study has underscored the impact of NPTMs before surgery on prognosis, the significance of postoperative STMs remains underexplored. Recently, some researches have paid attention to the role of postoperative STMs and found that they are also promising indicators (9, 13, 14). It has also been shown that the number of positive tumour markers before and after treatment is important for the prognosis of rectal cancer (15, 16). Therefore, we believe that combining both preoperative and postoperative CEA and CA19-9 measures might enhance predictive accuracy.

While clinical predictive models are endorsed for estimating the recurrence and survival of diverse malignancies due to their utility and comprehensiveness (17, 18), no research has incorporated NPTMs before and after surgery into these models for stage I-III CRC. Recognizing the vital prognostic implications of NPTMs, we evaluated the association of preoperative and postoperative CEA and CA199 with OS and DFS in patients with stage I-III CRC who underwent radical resection. Age, sex, pN stage, NPTMs before and after surgery were chosen to construct the clinical prediction models of overall survival(OS) and disease-free survival(DFS). Additionally, we further compared the clinical value of these models with that of the AJCC 8th TNM stage.

## 2 Patients and methods

### 2.1 Study population

**Figure 1:**
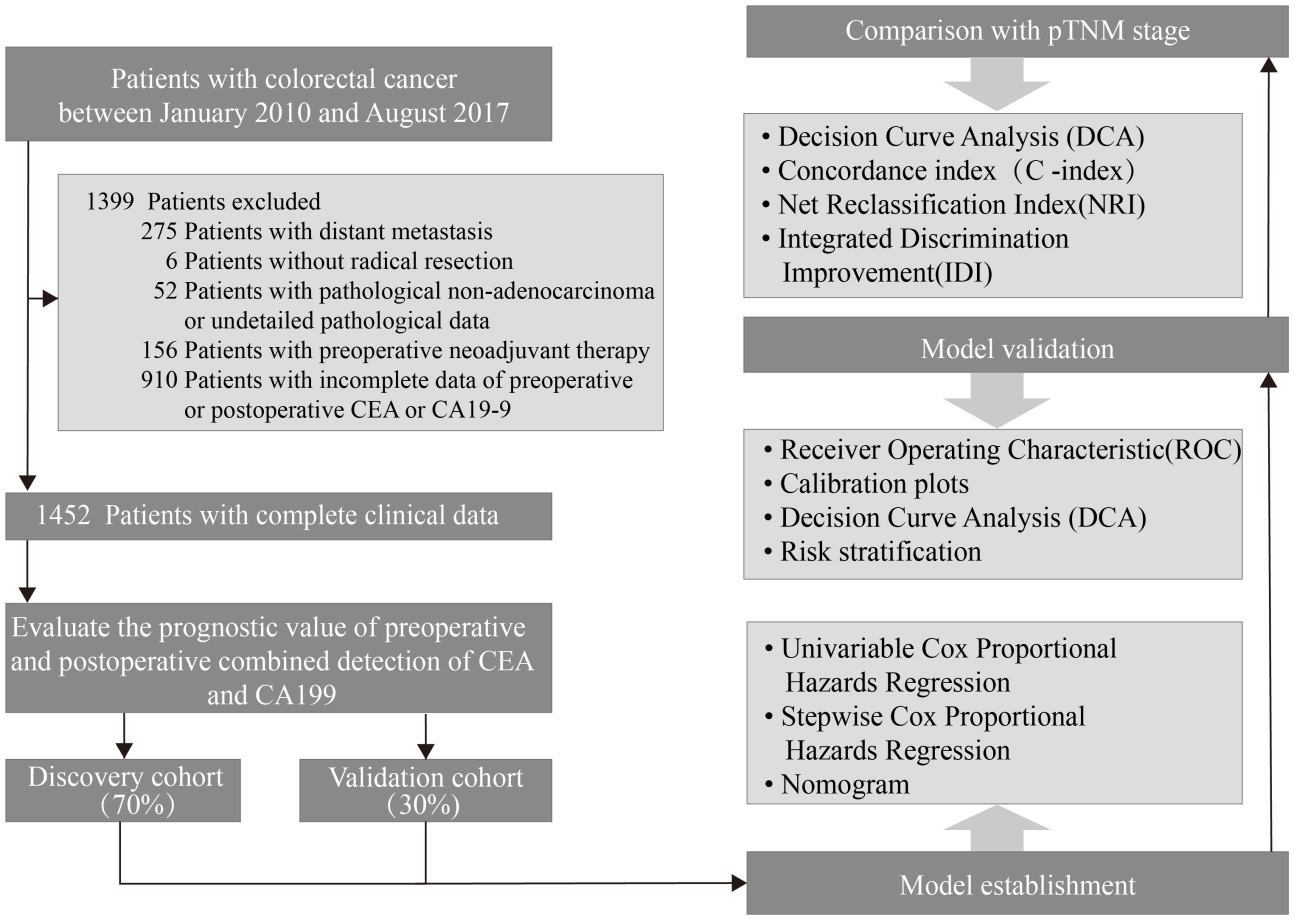
Study flow chart.

This study included consecutive CRC patients who underwent radical resection at the Department of Colorectal and Anal Surgery, Xinhua Hospital Affiliated to Shanghai Jiao Tong University School of Medicine from January 2010 to August 2017. Exclusion criteria were as follows (**Figure 1**): (1) patients with distant metastasis; (2) patients without radical resection; (3) patients with pathological non-adenocarcinoma or undetailed pathological data; (4) patients with preoperative neoadjuvant therapy; (5) patients with incomplete data of preoperative or postoperative CEA or CA19-9. Finally, 1,452 patients were involved in the study. The entire population was randomly grouped into a discovery cohort of 70% (n = 966) and a validation cohort of 30% (n = 486). All patients were staged according to the latest NCCN guidelines. All patients included in the study underwent radical (R0) resection of the primary tumour. Chemotherapy was administered according to NCCN guidelines to patients who met the criteria for postoperative chemotherapy.

### 2.2 Detection of CEA and CA19-9

Preoperative STMs (CEA, CA19-9) were tested within 7 days before radical surgery for colorectal cancer. Postoperative STMs (CEA, CA19-9) were tested in serum samples obtained at the patient’s first visit during the postoperative 2.5 − 3.5 months. A cutoff of 10 ng/ml was utilized to determine CEA positivity, while CA19-9 positivity was ascertained using a threshold of 39 U/ml (19–21). Patients were stratified based on the NPTMs before and after surgery as follows: (1) NPTMs was zero (both CEA and CA19-9 negative); (2) NPTMs was one (either CEA or CA19-9 positive); and (3) NPTMs was two (both CEA and CA19-9 positive). Patients were categorized based on NPTMs, followed by an analysis of their clinical characteristics and survival outcomes.

### 2.3 Follow-up study

Follow-up evaluations were conducted quarterly for the first two years after surgery. Subsequent assessments occurred biannually from the third to the fifth year, and then annually thereafter. In both cohorts, the follow-up protocol included physical examination, chest CT scan, measurement of CEA and CA19-9, abdominal and pelvic MRI or CT, etc. Colonoscopy was carried out once a year. OS is the time from radical resection to either death from any cause or the last follow-up, while DFS spans from radical resection to the first recurrence, any cause of death, or the last follow-up. The follow-up evaluation of this study concluded on August 2022.

### 2.4 Data analysis

The χ2 test or Fisher’s exact test was utilized to compare categorical variables. The Kaplan-Meier method and the log-rank test were employed, so as to assess the survival curves across groups. In the discovery cohort, traditional clinicopathological variables underwent the univariate analysis. Factors with P < 0.2 were incorporated as independent variables into the COX regression for a multivariate assessment. Variables were selected for inclusion in the nomograms based on the Akaike information criterion (AIC). Until the optimal model was obtained, AIC (Akaike information criterion, a standard for measuring statistical model fitting) was gradually reduced. The model with the lowest AIC value is usually chosen as the best model. The nomograms were used to predict the probability of survival and recurrence. The discrimination ability was evaluated by the concordance index (C-index) and receiver operating characteristic curve (ROC). The calibration curve was used to evaluate the calibration power. The net reclassification index (NRI) and integrated discrimination improvement (IDI) are designed to evaluate enhancements in risk forecasting and gauge the efficacy of the novel nomogram. They were used to compare the clinical value between nomograms and TNM stage. Decision curve analysis (DCA) is a method to evaluate the clinical applicability, quantifying its net benefit across various threshold probabilities. Curves representing all patients treated (indicating the highest clinical cost) and no treatment (indicating no clinical benefit) were used as references. All tests were conducted on both sides, with a significance level established at P<0.05. All data were analyzed using SPSS(26) and R software (4.2.1).

## 3 Result

### 3.1 Clinicopathological features

The study had 1,452 participants. The 5-year OS and DFS rates were 80.7% and 76.7%, respectively, with a median age of 63 years (IQR: 57 – 72 years). The discovery cohort included 966 cases, while the validation cohort had 486 cases (**Table 1**). The 5-year OS rates for the discovery and validation cohorts were 81.7% and 78.8%, respectively, while the 5-year DFS rates were 78.0% and 74.0%. In the discovery cohort, there were 531 men and 435 women. According to the TNM staging system, stages I, II, and III included 169 (17.5%), 395 (40.9%), and 402 (41.6%) cases, respectively. The age of participants in the validation cohort was obviously younger than that in the discovery cohort. (P < 0.05). Except for age, other variables showed no significant difference. (P > 0.05).

**Table 1:** Comparison of baseline clinicopathologic characteristics between the discovery cohort and the validation cohort.

Clinicopathological Features	Overall	Discovery cohort	Validation cohort	P value
	(n =1452)	(n = 966)	(n = 486)	
Sex				0.859
Male	795 (54.7)	531 (55.0)	264 (54.3)	
Female	657 (45.3)	435 (45.0)	222 (45.7)	
Age (years)				**0.019**
<65	790 (54.4)	504 (52.2)	286 (58.8)	
≥65	662 (45.6)	462 (47.8)	200 (41.2)	
Tumour Location				0.615
Right Colon	328 (22.6)	222 (23.0)	106 (21.8)	
Left Colon	445 (30.6)	288 (29.8)	157 (32.3)	
Rectum	679 (46.8)	456 (47.2)	223 (45.9)	
Histologic type				0.955
Grade I Adenocarcinoma	45 (3.1)	14 (2.9)	31 (3.2)	
Grade II Adenocarcinoma	1056 (72.7)	356 (73.3)	700 (72.5)	
Grade III Adenocarcinoma	131 (9.0)	45 (9.3)	86 (8.9)	
Mucinous Adenocarcinoma	220 (15.2)	71 (14.6)	149 (15.4)	
pT stage				0.353
T1	87 (6.0)	52 (5.4)	35 (7.2)	
T2	246 (16.9)	158 (16.4)	88 (18.1)	
T3	810 (55.8)	543 (56.2)	267 (54.9)	
T4	309 (21.3)	213 (22.0)	96 (19.8)	
pN stage				0.533
N0	849 (58.5)	564 (58.4)	285 (58.6)	
N1	364 (25.1)	249 (25.8)	115 (23.7)	
N2	239 (16.5)	153 (15.8)	86 (17.7)	
pTNM stage				0.48
I	266 (18.3)	169 (17.5)	97 (20.0)	
II	583 (40.2)	395 (40.9)	188 (38.7)	
III	603 (41.5)	402 (41.6)	201 (41.4)	
Perineural/Vascular invasion				0.59
No	1332 (91.7)	883 (91.4)	449 (92.4)	
Yes	120 (8.3)	83 (8.6)	37 (7.6)	
NPTMs before surgery				0.167
0	1062 (73.1)	696 (72.0)	366 (75.3)	
1	300 (20.7)	213 (22.0)	87 (17.9)	
2	90 (6.2)	57 (5.9)	33 (6.8)	
NPTMs after surgery				0.83
0	1346 (92.7)	897 (92.9)	449 (92.4)	
1	86 (5.9)	55 (5.7)	31 (6.4)	
2	20 (1.4)	14 (1.4)	6 (1.2)	

NPTMs, the number of positive tumour markers.

Statistically significant values are in bold.

### 3.2 Clinicopathological features based on preoperative and postoperative tumour markers

**Table 2** summarizes the association between NPTMs and the characteristics of patients. Preoperatively, 1,062 patients (73.1%) were negative for both markers, 300 patients (20.7%) were positive for one marker, and 90 patients (6.2%) were positive for both markers. Postoperatively,1,346 patients (92.7%) were negative for both markers, 86 (5.9%) patients were positive for one marker, and 20 (1.4%) patients were positive for both markers. There was a significant correlation between NPTMs before surgery and tumour location, histological type, pT stage, pN stage, pTNM stage, and nerve/vascular invasion (all P < 0.05; **Table 2**). The NPTMs after surgery was also significantly associated with age, histological type, pN stage, pTNM stage, and nerve/vascular invasion (all P < 0.05; **Table 2**).

**Table 2:** Associations of NPTMs with clinicopathological characteristics in stage I-III CRC patients after radical resection.

Clinicopathological Features	NPTMs before surgery			P value	NPTMs after surgery			P value
	0 (n = 1062)	1 (n = 300)	2 (n = 90)		0 (n = 1346)	1 (n = 86)	2 (n = 20)	
Sex				0.263				0.44
Male	590 (55.6)	163 (54.3)	42 (46.7)		739 (54.9)	43 (50)	13 (65)	
Female	472 (44.4)	137 (45.7)	48 (53.3)		607 (45.1)	43 (50)	7 (35)	
Age (years)				0.649				**0.011**
<65	570 (53.7)	169 (56.3)	51 (56.7)		747 (55.5)	34 (39.5)	9 (45)	
≥65	492 (46.3)	131 (43.7)	39 (43.3)		599 (44.5)	52 (60.5)	11 (55)	
Tumour Location				**0.003**				0.642
Right Colon	231 (21.8)	67 (22.3)	30 (33.3)		302 (22.4)	19 (22.1)	7 (35)	
Left Colon	308 (29)	112 (37.3)	25 (27.8)		417 (31)	24 (27.9)	4 (20)	
Rectum	523 (49.2)	121 (40.3)	35 (38.9)		627 (46.6)	43 (50)	9 (45)	
Histologic type				**< 0.001**				**0.025**
Grade I Adenocarcinoma	43 (4)	2 (0.7)	0 (0)		43 (3.2)	2 (2.3)	0 (0)	
Grade II Adenocarcinoma	791 (74.5)	200 (66.7)	65 (72.2)		987 (73.3)	59 (68.6)	10 (50)	
Grade III Adenocarcinoma	80 (7.5)	38 (12.7)	13 (14.4)		112 (8.3)	13 (15.1)	6 (30)	
Mucinous Adenocarcinoma	148 (13.9)	60 (20)	12 (13.3)		204 (15.2)	12 (14)	4 (20)	
pT stage				**< 0.001**				0.079
T1	82 (7.7)	3 (1)	2 (2.2)		80 (5.9)	7 (8.1)	0 (0)	
T2	214 (20.2)	26 (8.7)	6 (6.7)		237 (17.6)	8 (9.3)	1 (5)	
T3	580 (54.6)	183 (61)	47 (52.2)		751 (55.8)	48 (55.8)	11 (55)	
T4	186 (17.5)	88 (29.3)	35 (38.9)		278 (20.7)	23 (26.7)	8 (40)	
pN stage				**< 0.001**				**< 0.001**
N0	678 (63.8)	136 (45.3)	35 (38.9)		798 (59.3)	47 (54.7)	4 (20)	
N1	244 (23)	87 (29)	33 (36.7)		344 (25.6)	16 (18.6)	4 (20)	
N2	140 (13.2)	77 (25.7)	22 (24.4)		204 (15.2)	23 (26.7)	12 (60)	
pTNM stage				**< 0.001**				**0.009**
I	237 (22.3)	23 (7.7)	6 (6.7)		252 (18.7)	14 (16.3)	0 (0)	
II	441 (41.5)	113 (37.7)	29 (32.2)		546 (40.6)	33 (38.4)	4 (20)	
III	384 (36.2)	164 (54.7)	55 (61.1)		548 (40.7)	39 (45.3)	16 (80)	
Perineural/Vascular invasion				**0.041**				**0.01**
No	986 (92.8)	266 (88.7)	80 (88.9)		1242 (92.3)	75 (87.2)	15 (75)	
Yes	76 (7.2)	34 (11.3)	10 (11.1)		104 (7.7)	11 (12.8)	5 (25)	

NPTMs, the number of positive tumour markers.

Statistically significant values are in bold.

### 3.3 OS and DFS based on preoperative and postoperative tumour markers

**Figure 2:**
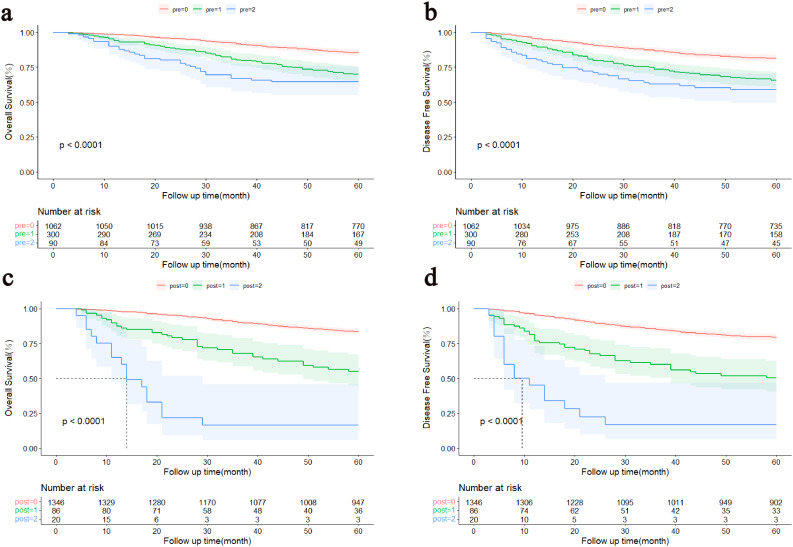
Kaplan–Meier curves of the patients with stage I-III colorectal cancer. **(A)** Association between the NPTMs before surgery and OS. **(B)** Association between the NPTMs before surgery and DFS. **(C)** Association between the NPTMs after surgery and OS. **(D)** Association between NPTMs after surgery and DFS. OS, overall survival; DFS, disease-free survival; NPTMs, the number of positive tumour markers; pre, the number of positive tumour markers before surgery; post, the number of positive tumour markers after surgery.

Kaplan-Meier survival curve results displayed obvious decreases in 5-year survival with increasing NPTMs before surgery (5-year OS rate: n = 0: 85.1%; n = 1: 69.9%; n = 2: 64.4%, P < 0.0001, **Figure 2A**; 5-year DFS rate: n = 0: 81.3%; n = 1: 65.6%; n = 2: 59.0%, P < 0.001, **Figure 2B**); similarly, there was also a obvious correlation between the NPTMs after surgery and patients’ OS and DFS (5-year OS rate: n = 0: 83.3%; n = 1: 54.9%; n = 2: 16.9%, P < 0.0001, **Figure 2C**; 5-year DFS rate: n = 0: 79.2%; n = 1: 50.3%; n = 2: 116.4%, P < 0.001, **Figure 2D**).

### 3.4 Nomogram variable screening

**Tables 3** and **4** showed the consequences of the variables analyses concerning survival in CRC patients. Multivariate COX regression analysis showed that sex, age, pN stage, NPTMs before and after surgery were independent prognostic factors for OS (**Table 3**); sex, age, pN stage, NPTMs before and after surgery were also independent prognostic factors for DFS (**Table 4**).

**Table 3:** Univariate and multivariate COX analysis of clinicopathological characteristics concerning overall survival of CRC patients in the discovery cohort.

Clinicopathological Features	Univariable analysis			Multivariable analysis		
	HR	95%CI	P value	HR	95%CI	P value
Sex						
Male	Reference					
Female	0.646	0.471 - 0.887	**0.007**	0.583	0.421 - 0.807	**0.001**
Age (years)						
<65	Reference					
≥65	2.045	1.490 - 2.807	**0.000**	2.424	1.741 - 3.374	**0.000**
Tumour Location						
Right Colon	Reference		** **			** **
Left Colon	0.553	0.358 - 0.854	**0.008**			0.218
Rectum	0.863	0.603 - 1.234	0.420			0.449
Histologic type						
Grade I Adenocarcinoma	Reference					
Grade II Adenocarcinoma	4.530	0.632 - 32.463	0.133			0.372
Grade III Adenocarcinoma	12.624	1.717 - 92.783	**0.013**			0.091
Mucinous Adenocarcinoma	6.979	0.955 - 51.026	0.056			0.199
pN stage						
N0	Reference					
N1	2.301	1.572 - 3.368	**0.000**	2.262	1.538 - 3.326	**0.000**
N2	5.195	3.600 - 7.496	**0.000**	4.627	3.148 - 6.801	**0.000**
NPTMs before surgery						
0	Reference		** **			
1	2.114	1.508 - 2.965	**0.000**	1.573	1.105 - 2.239	**0.012**
2	3.611	2.247 - 5.803	**0.000**	2.550	1.495 - 4.348	**0.001**
NPTMs after surgery						
0	Reference		** **			
1	2.901	1.814 - 4.640	**0.000**	1.789	1.078 - 2.967	**0.024**
2	12.447	6.521 - 23.761	**0.000**	4.187	2.038 - 8.602	**0.000**

NPTMs, the number of positive tumour markers.

Statistically significant values are in bold.

**Table 4:** Univariate and multivariate COX analyses of clinicopathological characteristics concerning disease-free survival of CRC patients.

Clinicopathological Features	Univariable analysis			Multivariable analysis		
	HR	95%CI	P value	HR	95%CI	P value
Sex						
Male	Reference					
Female	0.820	0.620 - 1.084	0.163	0.739	0.556 - 0.984	**0.038**
Age (years)						
<65	Reference					
≥65	1.937	1.457 - 2.573	**0.000**	2.235	1.661 - 3.007	**0.000**
Tumour Location						
Right Colon	Reference					
Left Colon	0.589	0.397 - 0.873	**0.008**			0.287
Rectum	0.908	0.655 - 1.260	0.564			0.268
Histologic type						
Grade I Adenocarcinoma	Reference					
Grade II Adenocarcinoma	1.423	0.526 - 3.848	0.487			0.867
Grade III Adenocarcinoma	3.289	1.159 - 9.335	**0.025**			0.305
Mucinous Adenocarcinoma	2.072	0.740 - 5.799	0.165			0.620
pN stage						
N0	Reference					
N1	1.833	1.302 - 2.582	**0.001**	1.856	1.312 - 2.627	**0.000**
N2	4.281	3.090 - 5.930	**0.000**	4.012	2.843 - 5.663	**0.000**
NPTMs before surgery						
0						
1	1.826	1.339 - 2.490	**0.000**	1.431	1.036 - 1.978	**0.030**
2	2.962	1.896 - 4.629	**0.000**	2.232	1.365 - 3.650	**0.001**
NPTMs after surgery						
0	Reference					
1	2.713	1.755 - 4.195	**0.000**	1.676	1.050 - 2.673	**0.030**
2	8.743	4.607 -16.594	**0.000**	2.986	1.478 - 6.031	**0.002**

NPTMs, the number of positive tumour markers.

Statistically significant values are in bold.

### 3.5 Construction and validation of nomograms for CRC

**Figure 3:**
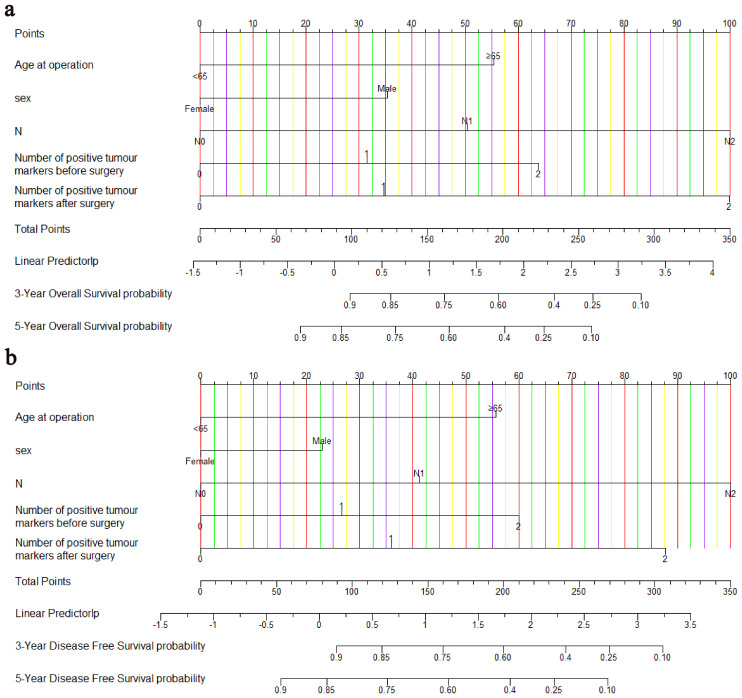
Nomograms for predicting OS **(A)** and DFS **(B)**. OS, overall survival; DFS, disease-free survival.

**Figure 4:**
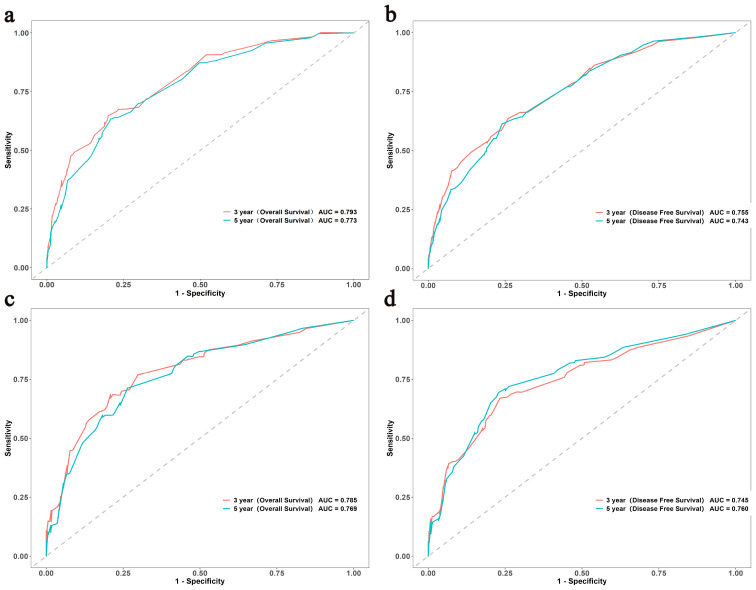
The ROC curves of the OS and DFS in both the discovery **(A, B)** and validation **(C, D)** cohorts. ROC, Receiver Operating Characteristic; OS, overall survival; DFS, disease-free survival.

Age, sex, pN stage, NPTMs before and after surgery were selected to construct nomograms for OS and DFS, respectively (**Figure 3**). For OS, the C-index was 0.760 for the discovery cohort and 0.772 for the validation cohort. For the discovery cohort, the model’s AUC values stood at 0.793 for 3 years and 0.773 for 5 years(**Figure 4A**). Concurrently, for the validation cohort, they were 0.785 and 0.769 (**Figure 4C**). For DFS, the C-index was 0.724 for the discovery cohort and 0.748 for the validation cohort. For the discovery cohort, the model’s AUC values stood at 0.755 for 3 years and 0.743 for 5 years (**Figure 4B**). Concurrently, for the validation cohort, they were 0.745 and 0.760 (**Figure 4D**). In addition, the calibration curves exhibited strong concordance between the models’ predictions and actual observations in both cohorts (**Supplementary Figure 1**).

### 3.6 Clinical value of nomograms compared with TNM stage

**Figure 5:**
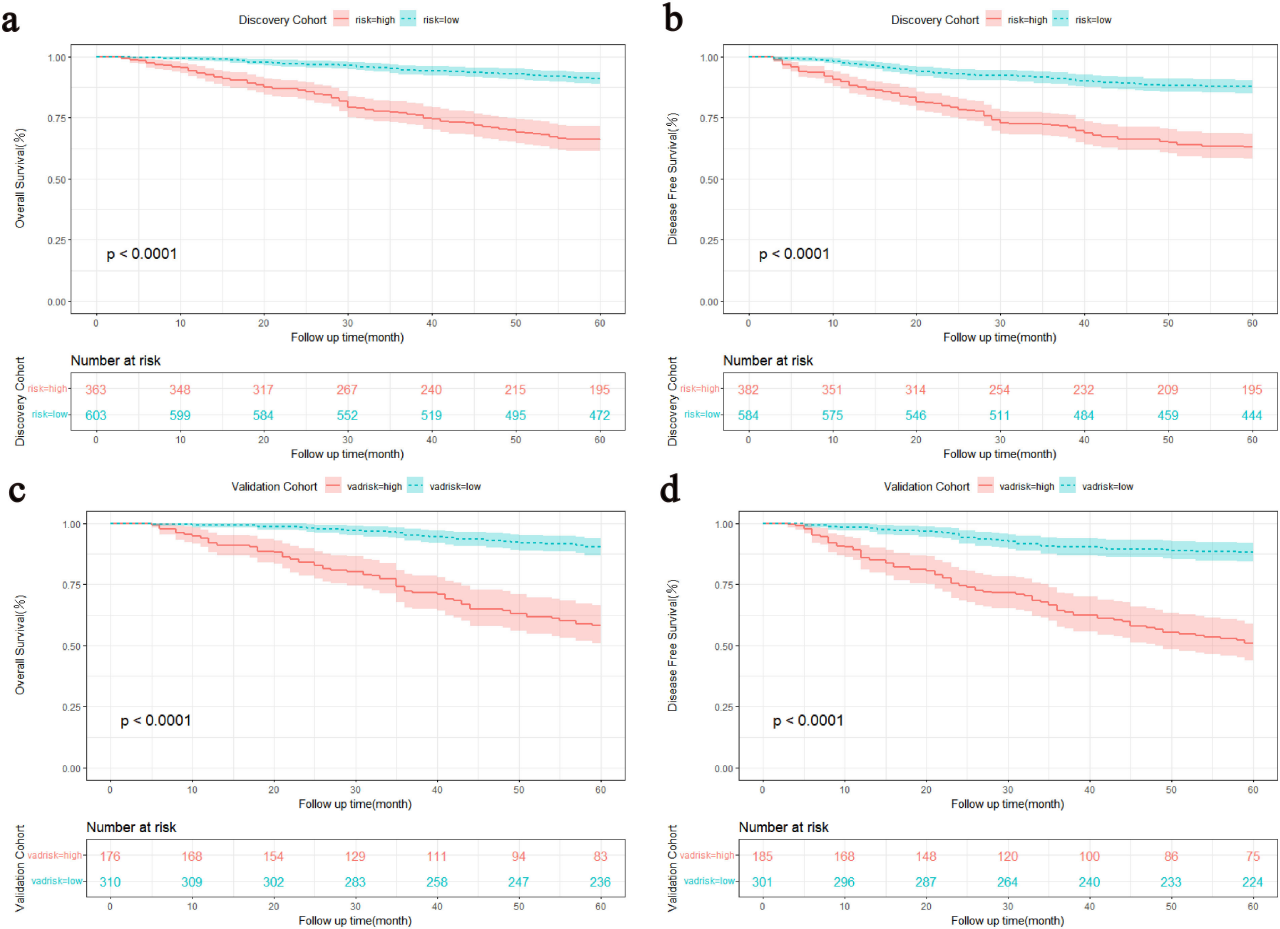
Kaplan-Meier survival curves stratified by the median of the total score of the discovery cohort. **(A, C)** OS curves for the discovery and validation cohorts. **(B, D)** DFS curves for the discovery and validation cohorts. OS, Overall survival; DFS, Disease-free survival.

The DCA showed that the nomograms offered superior net clinical benefits for both OS and DFS compared with TNM stage (**Supplementary Figure 2**). To further compare the accuracy of the models with the conventional TNM stage, we also analyzed the C-index change, NRI, and IDI (**Table 5**). Within the discovery cohort, the C-index change for OS was 0.105, The NRI for OS at 3 and 5 years registered at 0.519 and 0.515, respectively. IDI was 0.131 and 0.117; The C-index change for DFS was 0.090, and the NRI for DFS at 3 and 5 years registered at 0.481 and 0.444, respectively. IDI was 0.101 and 0.098, respectively. This result was further verified in the validation cohort. In addition, Participants were grouped into two different risk groups based on the median of the risk group scores in the discovery cohort. Results from the Kaplan-Meier survival curves revealed notable distinctions between two different risk cohorts (P < 0.01, **Figure 5**). Overall, our nomograms demonstrated superior predictive performance and clinical applicability compared with the traditional TNM stage, offering a more precise prognosis and survival prediction for patients.

**Table 5:** Comparison between nomograms and pTNM stage in C-index, NRI, and IDI.

Index	Discovery cohort			Validation cohort		
	Estimate	95% CI	P value	Estimate	95% CI	P value
NRI (vs.pTNM stage)						
For 3-year OS	0.519	0.389 - 0.653		0.464	0.298 - 0.627	
For 5-year OS	0.515	0.378 - 0.646		0.472	0.308 - 0.635	
IDI (vs.pTNM stage)						
For 3-year OS	0.131	0.087 - 0.197	0.000	0.172	0.095 - 0.273	0.000
For 5-year OS	0.117	0.083 - 0.172	0.000	0.140	0.088 - 0.217	0.000
C-index (OS)						
The nomogram	0.760	0.724 - 0.796		0.772	0.723 - 0.821	
The pTNM stage	0.655	0.618 - 0.691		0.665	0.619 - 0.711	
Change	0.105	0.064 - 0.142	0.000	0.107	0.056 - 0.165	0.000
NRI (vs.pTNM stage)						
For 3-year DFS	0.481	0.344 - 0.587		0.423	0.243 - 0.648	
For 5-year DFS	0.444	0.307 - 0.555		0.406	0.240 - 0.580	
IDI (vs.pTNM stage)						
For 3-year DFS	0.101	0.071 - 0.149	0.000	0.126	0.080 - 0.206	0.000
For 5-year DFS	0.098	0.066 - 0.145	0.000	0.109	0.061 - 0.186	0.000
C-index (DFS)						
The nomogram	0.724	0.689 - 0.759		0.748	0.701 - 0.795	
The pTNM stage	0.633	0.600 - 0.667		0.676	0.635 - 0.716	
Change	0.090	0.051 - 0.132	0.000	0.072	0.034 - 0.112	0.000

## 4 Discussion

Researches on biomarkers of gastrointestinal cancer have been widely concerned. A study on dogs has explored the important role of lipopolysaccharide in intestinal carcinogenesis (22), and Li et al.’ s study on mice suggested that the secretory protein cathepsin K can be used as a new predictive biomarker for CRC (23). In addition, previous studies have confirmed the potential prognostic value of absolute quantification of free circulating DNA (24)and long non-coding RNA plasmacytoma variant translocation 1 (25) in CRC patients as biomarkers. Serum CEA and CA19-9 are common and cost-effective biomarkers in clinical practice and they are instrumental in predicting the prognosis of CRC, holding significant value in both pre- and post-operation (26, 27). However, previous researches have primarily paid attention to the prognostic significance of preoperative STMs (28–30), with little attention given to postoperative CEA and CA19-9. Study has demonstrated that in terms of predicting survival duration, combined tumour markers assessments hold an advantage over single marker tests (31). In recent years, NPTMs, introduced as a novel reference index, has exhibited profound prognostic potential (11, 15). Given their significant clinical value, this study categorized patients according to NPTMs, assessed the prognostic significance of combined STMs detection before and after surgery, and subsequently developed clinical prediction models.

In this study, we found that the increased NPTMs before and after surgery were associated with a poor prognosis of CRC. Furthermore, NPTMs before surgery was closely related to the TNM stage and tumour location, consistent with prior findings (11). Studies reported that patients with normal STMs after surgery possessed a notably better prognosis compared with patients with abnormal STMs (13, 15). This research also supported this result. Compared with patients with normal postoperative CEA and CA19-9, patients with both tumour markers positive postoperatively had approximately a 4.2-fold increased risk of death and a 3.0-fold increased risk of recurrence. We also discovered that both preoperative and postoperative positive CEA and CA19-9 were more likely to occur in population with higher pTNM stage, higher pN stage, and those with neural/vascular invasion. For these patients, a more intensive follow-up strategy should be implemented.

The role of circulating tumour DNA (ct-DNA) in predicting the prognosis of colorectal cancer has garnered widespread attention (32, 33). Study has reported a correlation between CT-DNA in tumour cells and residual microcancer cells, but its clinical application remains limited due to its high costs (34). Conversely, tumour marker detection is affordable and easy to operate. Konishi et al. reported that patients with elevated postoperative CEA faced a higher hazard of early recurrence, especially within the first year after radical surgery (13). Sonoda et al. found that elevation of CEA post-surgery is independently correlated with an unfavorable prognosis in stage II-III CRC (14). In this study, we found that NPTMs before and after surgery were independent prognostic factors for OS and DFS in patients with stage I-III colorectal cancer. The elevation of tumour marker levels postoperatively may suggest the presence of unrecognized residual minute cancer cells at the time of surgery or in postoperative radiological examinations, which raises the possibility of relapse (35, 36). Therefore, in clinical practice, it is essential not only to perform combined tumour markers testing before surgery for colorectal cancer patients but also to pay attention to postoperative combined tumour markers testing. Patients with positive tumour markers might benefit from comprehensive treatment and require followed-up regularly.

TNM stage is commonly used for prognosis prediction and assessment, but its ability to predict patient outcomes may be limited (37). Nomogram is a powerful graphical prediction tool that illustrates the likelihood of a specific event occurring based on multiple variables (17). Compared with the TNM stage, the nomogram is more intuitive and easier to understand. Moreover, it can incorporate more risk factors, significantly enhancing the accuracy of prediction. Previous studies have developed CRC-related survival prediction models based on STMs (38, 39), but they were limited in sample size and did not focus on the prognostic significance of post-surgical STMs. This might have restricted their predictive accuracy to some extent. Therefore, this study constructed two more comprehensive clinical prediction models based on NPTMs to help clinicians predict and evaluate the prognosis. Both nomograms included five variables: sex, age at surgery, pN stage, NPTMs before and after surgery. The pN stage and NPTMs after surgery had significant effects on total scores of two models.

The C-index results indicated that the discriminative ability of the two models is significantly superior to the TNM stage. Risk stratification analysis revealed that the predictive models for OS and DFS exhibited commendable discrimination proficiency. Further, the results of DCA underscored that our models exhibited superior performance in clinical decision-making compared with TNM stage. The calibration curves for both groups also confirmed the strong concordance between the predictive model and the actual outcomes. NRI and IDI are two statistical indicators used to assess the enhanced performance of predictive models. Through their comparative analysis, we can discern the differential performance of various models and select the optimal one (40, 41). Within our investigation, both NRI and IDI metrics indicated that the novel models had superior accuracy and discriminatory ability in forecasting 3-year and 5-year OS and DFS for CRC patients. To summarize, both prognostic models exhibited strong predictive efficacy and clinical applicability, and can be utilized in clinical settings to forecast the prognosis of stage I-III CRC patients.

This study had several limitations. First of all, both the discovery cohort and validation cohort were established through random grouping, which could lead to imbalances at baseline. For instance, distinct variations in clinicopathological characteristics were observed in both cohorts (age, P < 0.05). Secondly, for the constructed model, the validation cohort performed better than the discovery cohort in some aspect. Thirdly, since stage IV patients receive different treatment methods from stage I–III patients, we did not include stage IV patients in our study, so the nomograms cannot be applied to stage IV CRC patients. Finally, Some factors that might be associated with prognosis, such as BRAF and KRAS mutation status and nutritional condition, were not considered in our study. Future studies should incorporate more valuable prognostic factors and conduct external validation.

## 5 Conclusion

NPTMs, both preoperatively and postoperatively, were closely related to the prognosis of stage I-III colorectal cancer patients. Compared with the AJCC 8th TNM stage, the nomograms based on preoperative and postoperative CEA and CA19-9 demonstrated superior predictive capability and clinical applicability, offering more precise prognosis for colorectal cancer patients. The results of this study suggest that preoperative and postoperative CEA and CA199, are crucial in predicting patients’ prognosis, and both clinicians and patients should be aware of the importance of the preoperative and postoperative testing of these two tumour markers. Therefore, it is not recommended to ignore the testing of these markers for various reasons. Further validation of the nomograms in different cohorts is needed to enhance their generalizability.

## Data Availability

The data presented in this study are available on reasonable request from the corresponding author. The data are not publicly available due to privacy.

## Supplementary material

Supplementary Figure 1 The calibration curves for forecasting the survival of the discovery cohort and validation cohort. **(A, C)** Calibration curves for the OS of the discovery and validation cohorts. **(B, D)** Calibration curves for the DFS of the discovery and validation cohorts. OS, overall survival; DFS, disease-free survival. A model's predicted probability or score is represented on the x-axis. This is the model's estimate of how likely an event is to occur. In addition, the y-axis shows the rate of event occurrence, also in the range 0 to 1. This is the proportion of events that occur in real data. Using the calibration curve, we can plot the relationship between the predicted probabilities of the model and the actual observations. A dotted line represents the ideal calibration line of the theory, which is 45 degrees diagonally. When the calibration curve coincides with this line, the model makes perfect predictions. Error lines (yellow and blue) on the calibration curve indicate its uncertainty. Usually, error lines represent confidence intervals.

Supplementary Figure 2 DCA of the nomogram and the AJCC 8th TNM stage for the survival prediction. **(A, C)** DCA for 3-year OS in the discovery and validation cohorts. **(B, D)** DCA for 5-year OS in the discovery and validation cohorts. **(E, G)** DCA for 3-year DFS in the discovery and validation cohorts. **(F, H)** DCA for 5-year DFS in the discovery and validation cohorts. DCA, Decision Curve Analysis; OS, overall survival; DFS, disease-free survival. By quantifying the net benefit at different threshold probabilities, DCA helps evaluate the clinical utility of a model. As benchmarks, we used curves representing full treatment, denoting maximum clinical benefits, and no treatment, denoting no clinical benefits.
