# Supplementary figures and images for: Development and validation of nomograms based on pre-/post-operative CEA and CA19-9 for survival predicting in stage I-III colorectal cancer patients after radical resection
Source: Front Oncol. 2024 Oct 11;14:1402847. doi: 10.3389/fonc.2024.1402847 (PMC11502300; doi:10.3389/fonc.2024.1402847)

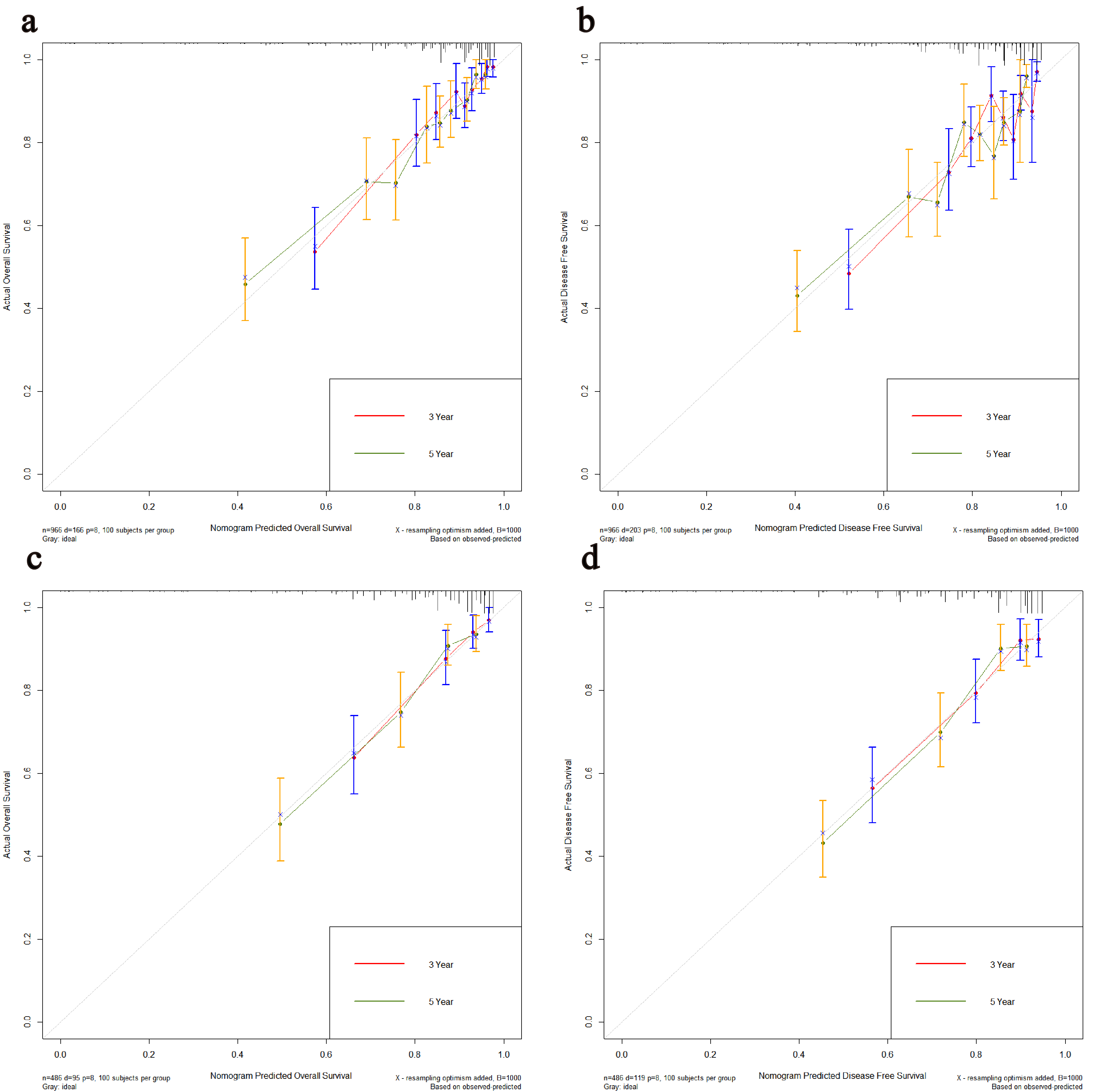

Supplement: Supplementary Figure 1 — The calibration curves for forecasting the survival of the discovery cohort and validation cohort. (A, C) Calibration curves for the OS of the discovery and validation cohorts. (B, D) Calibration curves for the DFS of the discovery and validation cohorts. OS, overall survival; DFS, disease-free survival. A model's predicted probability or score is represented on the x-axis. This is the model's estimate of how likely an event is to occur. In addition, the y-axis shows the rate of event occurrence, also in the range 0 to 1. This is the proportion of events that occur in real data. Using the calibration curve, we can plot the relationship between the predicted probabilities of the model and the actual observations. A dotted line represents the ideal calibration line of the theory, which is 45 degrees diagonally. When the calibration curve coincides with this line, the model makes perfect predictions. Error lines (yellow and blue) on the calibration curve indicate its uncertainty. Usually, error lines represent confidence intervals. [file Image1.tif]

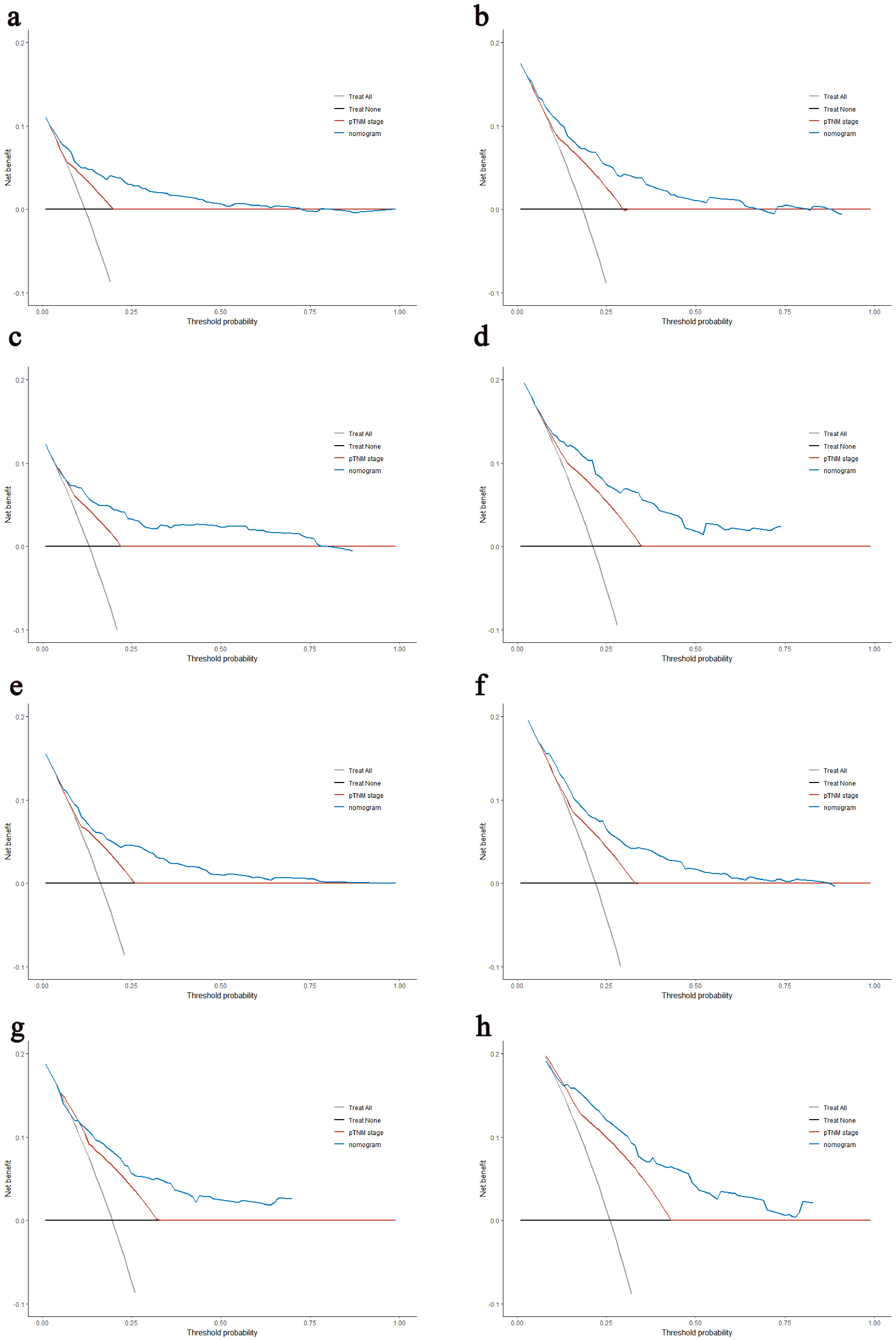

Supplement: Supplementary Figure 2 — DCA of the nomogram and the AJCC 8th TNM stage for the survival prediction. (A, C) DCA for 3-year OS in the discovery and validation cohorts. (B, D) DCA for 5-year OS in the discovery and validation cohorts. (E, G) DCA for 3-year DFS in the discovery and validation cohorts. (F, H) DCA for 5-year DFS in the discovery and validation cohorts. DCA, Decision Curve Analysis; OS, overall survival; DFS, disease-free survival. By quantifying the net benefit at different threshold probabilities, DCA helps evaluate the clinical utility of a model. As benchmarks, we used curves representing full treatment, denoting maximum clinical benefits, and no treatment, denoting no clinical benefits. [file Image2.tif]
